# Dissecting the EGFR-PI3K-AKT pathway in oral cancer highlights the role of the EGFR variant III and its clinical relevance

**DOI:** 10.1186/1423-0127-20-43

**Published:** 2013-06-27

**Authors:** Kwang-Yu Chang, Shan-Yin Tsai, Shang-Hung Chen, Hsiao-Hui Tsou, Chia-Jui Yen, Ko-Jiunn Liu, Hsun-Lang Fang, Hung-Chang Wu, Bin-Fay Chuang, Shao-Wen Chou, Careen K Tang, Shyun-Yeu Liu, Pei-Jung Lu, Ching-Yu Yen, Jang-Yang Chang

**Affiliations:** 1 Institute of Clinical Medicine College of Medicine, National Cheng Kung University, 7th Floor, No.35 Xiaodong Rd., Tainan City 701, Taiwan; 2 National Institute of Cancer Research National Health Research Institutes, 2nd Floor No.367, Shengli Rd., Tainan City 70456, Taiwan; 3 Institute of Population Health Sciences National Health Research Institutes, No.35 Keyan Road, Zhunan, Miaoli County 35053, Taiwan; 4 Division of Hematology/Oncology Department of Internal Medicine, National Cheng Kung University Hospital, No.138 Shengli Rd., Tainan 704, Taiwan; 5 Department of Pathology, Kaohsiung Medical University Hospital, No.100 , Tzyou 1st Rd., Kaohsiung 807, Taiwan; 6 Division of Hematology and Oncology, Department of Internal Medicine, Chi-Mei Medical Center, Liouying, No. 201, Taikang Village, Liuying Dist, Tainan City 73657, Taiwan; 7 Department of Cosmetology and Health Care, Min-Hwei College of Health Care Management, No.1116, Sec. 2, Zhongshan E. Rd., Liuying Dist., Tainan 73658, Taiwan; 8 Division of Hematology and Oncology, Department of Internal Medicine, Chi-Mei Medical Center, No.901, Zhonghua Rd., Tainan City, Yongkang Dist. 710, Taiwan; 9 Oral and Maxillofacial Surgery Section, Chi-Mei Medical Center, No.901, Zhonghua Rd., Tainan City, Yongkang Dist. 710, Taiwan; 10 Department of Oncology, Lombardi Comprehensive Cancer Center, Georgetown University Medical Center, 3800 Reservoir Rd., Washington, DC 20057, USA; 11 Department of Dentistry, National Defense Medical Center, No.161, Sec. 6, Minquan E. Rd., Taipei City, Neihu District 114, Taiwan; 12 Department of Dentistry, Taipei Medical University, No. 250, Wu-Hsing Street, Taipei City 110, Taiwan

**Keywords:** AKT, EGFR, EGFRvIII, Oral cancer, PI3K, PTEN

## Abstract

**Background:**

Dysregulated epidermal growth factor receptor (EGFR)-phosphoinositide-3-kinase (PI3K)-AKT signaling is considered pivotal for oral cancer, and the pathway is a potential candidate for therapeutic targeting.

**Results:**

A total of 108 archival samples which were from surgically resected oral cancer were examined. Immunohistochemical staining showed the protein expression of membranous wild-type EGFR and cytoplasmic phosphorylated AKT was detected in 63.9% and 86.9% of the specimens, respectively. In 49.1% of the samples, no phosphatase and tensin homolog (PTEN) expression was detected. With regard to the EGFR variant III (EGFRvIII), 75.0% of the samples showed positive expression for moderate to severe staining, 31.5% of which had high expression levels. Real-time polymerase chain reaction assays for gene copy number assessment of *PIK3CA* revealed that 24.8% of the samples had alterations, and of *EGFR* showed that 49.0% had amplification. Direct sequencing of *PIK3CA* gene showed 2.3% of the samples had a hotspot point mutation. Statistical assessment showed the expression of the EGFRvIII correlated with the T classification and TNM stage. The Kaplan-Meier analyses for patient survival showed that the individual status of phosphorylated AKT and EGFRvIII led to significant differences in survival outcome. The multivariate analysis indicated that phosphorylated AKT, EGFRvIII expression and disease stage were patient survival determinants.

**Conclusions:**

Aberrations in the EGFR-PI3K-AKT pathway were frequently found in oral cancers. EGFRvIII and phosphorylated AKT were predictors for the patient survival and clinical outcome.

## Background

Oral cancer (OC) is a subtype of head and neck cancer (HNC) that arises from the oral cavity, and squamous cell carcinoma is the most frequent histological type. In 2008, the worldwide estimated incidence was 263,900 cases, ranking 10th for male cancers [1]. In Taiwan, the age-standardized incidence rate was 11.3 per 100,000 of the male population [2]. Together with oropharynx and hypopharynx diseases, HNC was the 4th most frequent male cancer in Taiwan. The high incidence of HNC is a consequence of prevalent betel nut consumption, which is a major national health issue [3]. The substance is harmful to the oral mucosa, leading to inflammation and cell cycle alterations in normal keratinocytes that may eventually contribute to tumorigenesis [4]. In fact, betel quid has a higher tendency to induce oral leukoplakia and submucosal fibrotic changes compared with other habits, such as cigarette smoking and alcohol consumption [5].

In contrast to traditional cytotoxic agents, evolving modern oncology focuses on the intracellular signal transduction pathways that are critical for cancer development. One example is the epidermal growth factor receptor (EGFR), a tyrosine kinase receptor located at the cell membrane. Signals are transmitted through the protein from extracellular ligands to intracellular molecular cascades [6]. Several studies have emphasized the role of the EGFR in HNC, suggesting a dependence on the elicited signal [7]. Based on this concept, a monoclonal antibody, cetuximab, has been developed [8,9]. At present, the successful introduction of the drug offers treatment options for patients with late-stage HNC.

Following cetuximab, numerous novel specific inhibitors currently under investigation are expected as therapeutic drugs in the future [10]. Nevertheless, a successful preclinical study does not always ensure clinical efficacy. To overcome such difficulties, it is fundamental to understand the molecular aberrations present in cancer cells. It has been reported that in addition to wild-type EGFR (EGFRwt), a truncated-form mutant, type III variant (EGFRvIII), is also frequently observed [11]. The mutant receptor distinguished to its wild-type counterpart as missing of the extracellular domain 1 and 2, which is encoded by the segment in exon 2 to exon 7. Lacking of these amino acids leads to structural activated conformation of the protein, showing constitutively activation with preferred signaling through the phosphoinositide-3-kinase (PI3K)-AKT pathway [12]. In addition to the receptor tyrosine kinase, the oncogenic cascade is oppositely regulated by intracellular factors to activate AKT through phosphorylation, e.g. PI3K and phosphatase and tensin homolog (PTEN). In fact, aberrations in these individual components often lead to tumorigenesis, indicating their crucial roles in HNC [11,13-16]. These investigations, however, have often lacked analyses of the serial cascade members. It is therefore worthwhile to undertake a global study of the EGFR-PI3K-AKT pathway.

In this article, we focused on the regulatory factors of the EGFR-PI3K-AKT pathway. The study used archived clinical OC samples to determine the proportions of aberrant genes and proteins. The analyses further showed the activating factors in this pathway and their clinical relevance.

## Methods

### Ethics statement

All tumor materials used in this study were obtained from the tissue bank of Chi-Mei Medical Center Yongkang and Liouying branches. Under the regulatory rules of the local ethics committees, the tumor samples were collected for the tissue bank after the patient consent forms were signed and completed. The samples were de-identified before receipt; thus, no additional patient consent was required. The study protocol, which included archival tissue procurement, was approved by the Institutional Review Board from Chi-Mei Medical Center (IRB 10012-L05). Formalin-fixed paraffin-embedded (FFPE) OC samples were then prepared. All specimens were histologically confirmed to be squamous cell carcinomas. The date of diagnosis was restricted to prior to the year 2006, with the follow-up data up to February 7, 2012. All patients received surgical resection and other standard treatments following the guidelines developed by the local head and neck cancer committee. Clinical information including gender, age, stage [17], tumor origin, and history of unhealthy habits was also obtained.

### Immunohistochemical (IHC) staining of tumor tissue

Antibodies for PTEN (138G8, #9559) and phosphorylated AKT (pAKT, Ser 473, D9E, #4060) were purchased from Cell Signaling Technology (Danvers, MA, USA), and the wild-type EGFR antibody (EGFRwt, Novocastra™ RTU-EGFR-384) was obtained from Leica Microsystems (Milton Keynes, UK). The developed monoclonal antibody for EGFRvIII (4-5H), which has been described previously, was also used [18]. IHC staining was performed on 5-μm-thick FFPE sections mounted on frosted slides. For pAKT, PTEN and EGFRvIII, the tissues were first warmed at 60°C for 30 minutes. They were then deparaffinized in xylene, followed by hydration with ethanol at concentrations from 100%, 95%, 85% to 75%. Next, the endogenous peroxidase was quenched through incubation in hydrogen peroxide for 15 minutes. For antigen retrieval, the samples were treated with autoclave boiling for 10 minutes in buffer. The sections were then incubated with specific antibodies in diluent at 4°C overnight. Next, a tag-staining procedure was applied to label the targets using the EnVision + ® kit (Dako, Glostrup, Denmark) according to the manufacturer’s protocol. For EGFRwt staining, the procedures were performed with the Bond-Max Automated IHC staining system (Leica Biosystems Newcastle Ltd, Australia) following the manufacturer’s standard protocol with minimal modifications. The slides were counterstained with hematoxylin.

### Interpretation of the IHC staining results

The intensity and percentage of the markers for each specimen were assessed by two independent pathologists. The criterion for positive staining was defined as more than 5% membranous staining for EGFRwt and more than 5% cytoplasmic staining for pAKT and PTEN. The percentage of EGFRvIII staining for each tumor specimen was classified into five staining groups according to the extent of moderate to strong cytoplasmic reactivity: 0, (none); 1+, (1 ~ 24%); 2+, (25 ~ 49%); 3+, (50 ~ 74%); and 4+, (75 ~ 100%).

### DNA sequencing for *PIK3CA* mutation

The entire genomic DNA was extracted from FFPE tissue using the Wizard® Genomic DNA Purification Kit (Promega, Madison, WI, USA) following the manufacturer’s protocol. *PIK3CA*, which encodes the catalytic subunit of class 1 PI3K, was highlighted because missense mutations are often found in cancer at G1624, G1633 in exon 9 and A3140 in exon 20 [19]. Mutations in these two exons which located in the helical domain and the kinase domain, respectively, led to an increased lipid kinase activity. For detection, specific primers for *PIK3CA* were added to the DNA for use with a PCR kit (Viogene, Taipei, Taiwan); the primers included the following sequences: exon 9 forward, 5-ccagaggggaaaaatatgaca-3, reverse, 5-cattttagcacttacctgtgac-3; and exon 20 forward, 5-catttgctccaaactgacca-3, reverse, 5-tgagctttcattttctcagttatcttttc-3 [20]. The amplified product was then sequenced for hotspot mutations using ABI Prism 3730 with the forward primers or the reverse primers, if necessary.

### Analysis of *PIK3CA* and *EGFR* copy numbers

The FAM™-labeled probes and the primers for *PIK3CA* and *EGFR* were purchased from Applied Biosystems (Foster City, CA, USA). The sequences used for gene copy analysis of *EGFR* were as follows: forward primer, 5-actggaaaaaactgtttgggacct-3; reverse primer, 5-agctgttttcacctctgttgcttat-3; and probe, 5-ccggtcagaaaacca-3 [21]. The primers and probe for the *PIK3CA* exon 20 were designed using TaqMan® Copy Number Variation Assay search tool on the Applied Biosystems website. The materials were then mixed with VIC® dye label-based RNase P for reference gene detection, the genomic DNA extraction and the Genotyping Master Mix (Applied Biosystems). Mononuclear cells from healthy donors were used for data normalization. For analysis, PCR was performed using the Applied Biosystems 7500 Fast Real-Time PCR System, and the cycle threshold (C_t_) was calculated. Copy number was assessed using the 2^-***ΔΔ***Ct^ method [22], with the normal gene copy number (GCN) set as 2. The cutoff point for amplification was set as 3 instead of 4 because of the unavoidable interference from nearby non-tumor tissue [23].

### Statistical analyses

All data analyses were calculated using SPSS 14.0 (SPSS Inc., Chicago, IL, USA) or SAS software, version 9.1 (SAS Institute, Inc., Cary, NC, USA). Two-sided *P*-*values* less than 0.05 were considered significant. The associations between factors were evaluated using the chi-squared test or Fisher’s exact test when sample sizes were small. The sample endpoint was overall survival, defined as period from the date of operation to the documented expired date. Kaplan-Meier survival analyses were performed to compare the differences in overall survival between subgroups using the log-rank test. Univariate and multivariate analyses were performed to identify the possible variables related to overall survival. The hazard ratio (HR) and corresponding 95% confidence interval (CI) on univariate and multivariate analyses were calculated using the Cox proportional hazard model. Factors of interest with *P*-*values* less than 0.1 and biological factors with probable impact were considered to be potentially associated with survival. These factors were then explored through multivariate analyses using Cox proportional hazards regression with a stepwise selection method to assess significance [24].

## Results

### Oral cancer samples are prepared for analysis

**Figure 1:**
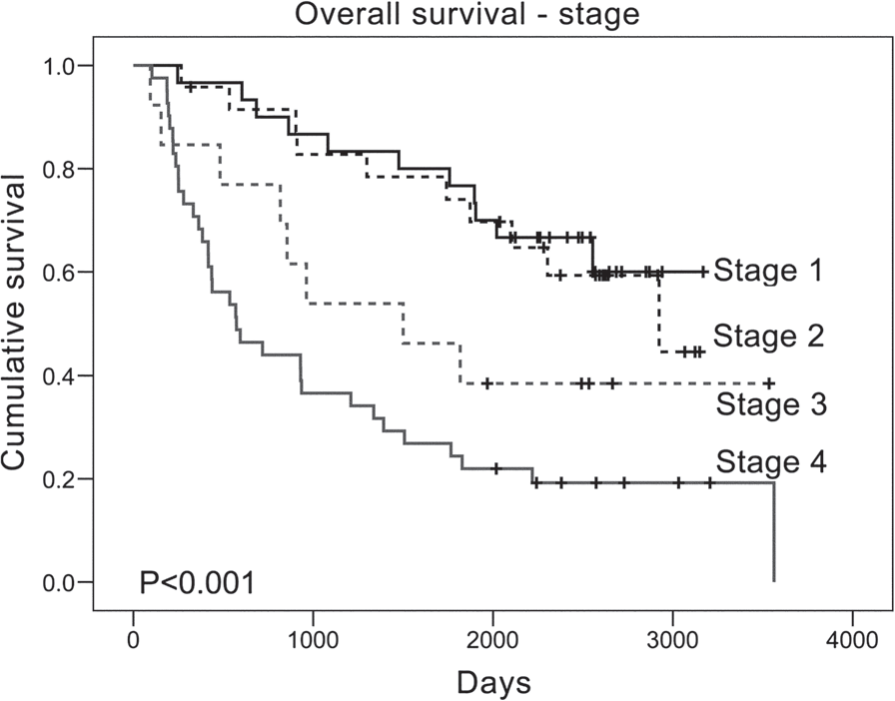
**Survival curves according to TNM stage.** Representative Kaplan-Meier plots for overall survival based on the classification of each stage. The vertical tick marks indicate censored data, including patients who were alive at the final day or who were lost to follow up.

Specimens from 108 patients were used; the demographic characteristics are listed in Table 1. In addition to surgery, 61.1% and 48.1% of the patients also received radiotherapy and chemotherapy, respectively. Up to 96.3% samples were from males. A total of 32.4% of the samples originated from the tongue, and 43.5% originated from buccal mucosa. The survival curve for each TNM stage is shown in Figure 1, with similar sample numbers in each group; however, there were relatively fewer samples in stage 3. The mean age was 50.6 years; the age distribution was normal and had a peak at the age group 41–50. Regarding patient habits, we found that 79.6% of the patients were either current or ever smokers and that 72.2% of the patients had experienced betel nut chewing. A total of 46.3% of the patients had a history of alcohol consumption. These data were typical for OC in Taiwan.

**Table 1:** **Demography of patients characteristic (*N*** **= 108)**

**Factors**	***N***	**Factors**	***N***
Origin		Age	
Buccal	47	≤30	2
Tongue	35	31-40	18
Gum	8	41-50	37
Lip	6	51-60	30
Palate	5	61-70	16
Retromolar trigone	4	≥71	5
Mouth floor	3	Smoking	
Gender		Ever	86
Male	104	Never	18
Female	4	Unknown	4
T classification		Alcohol	
1	38	Ever	50
2	36	Never	50
3	6	Unknown	8
4	28	Betel nut	
N classification		Ever	78
0	72	Never	22
1	15	Unknown	8
2	21	Radiation	
3	0	Received	66
TNM staging		Not received	33
1	30	Unknown	9
2	24	Chemotherapy	
3	13	Received	52
4	41	Not received	46
		Unknown	10

### Dysregulated expression of EGFRwt, EGFRvIII, PTEN, and pAKT is frequently observed

**Figure 2:**
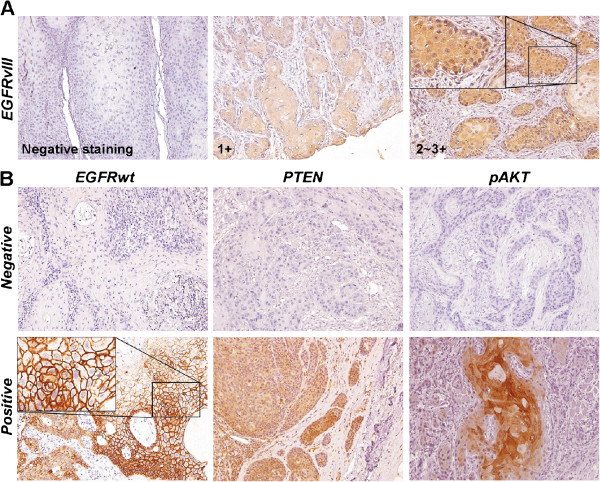
**Immunohistochemical staining of the tumor tissue.** (**A**) Negative EGFRvIII expression is shown on the left, grade 1 in the middle, and grade 2–3 in the right panels. The significant cytoplasmic staining is shown at a higher resolution in the inset square. (**B**) Representative EGFRwt, PTEN, and pAKT staining results are shown in the left, middle, and right columns, respectively. Examples of negative and positive samples are shown in the upper and lower rows, respectively. A higher resolution of EGFRwt membranous staining is shown in the inset square. Each digital image was directly captured to the computer using light microscopy at a resolution of 200 ×.

IHC staining was performed to detect EGFR, EGFRvIII and PTEN expression in all samples; however, one sample lacked pAKT staining due to an insufficient amount of tumor specimen. Regarding the subcelluar distribution, the majority of the immunoreactive signal was observed in the cytoplasm for EGFRvIII, PTEN and pAKT (Figure 2A, 2B). In contrast, EGFRwt expression was detected in both the membrane and the cytoplasm (Figure 2B). Using the criteria described in the Methods section, 49.1% of these specimens were negative for PTEN expression, and 86.9% were positive for pAKT expression. With regard to EGFRwt, 63.9% of the samples were positive for the membranous expression of the wild-type protein [25]. Conversely, moderate to strong levels of EGFRvIII staining were present in the cytoplasm of 75% of the samples. EGFRvIII staining was further scored as 1+, 2+, 3+, and 4+ based on the area extent, with 24.1%, 19.4%, 24.1%, and 7.4% of the samples in each category, respectively. In summary, the aberrant expression of the individual cascade members was frequently observed.

### EGFR amplification correlates with dysregulated protein expression

**Figure 3:**
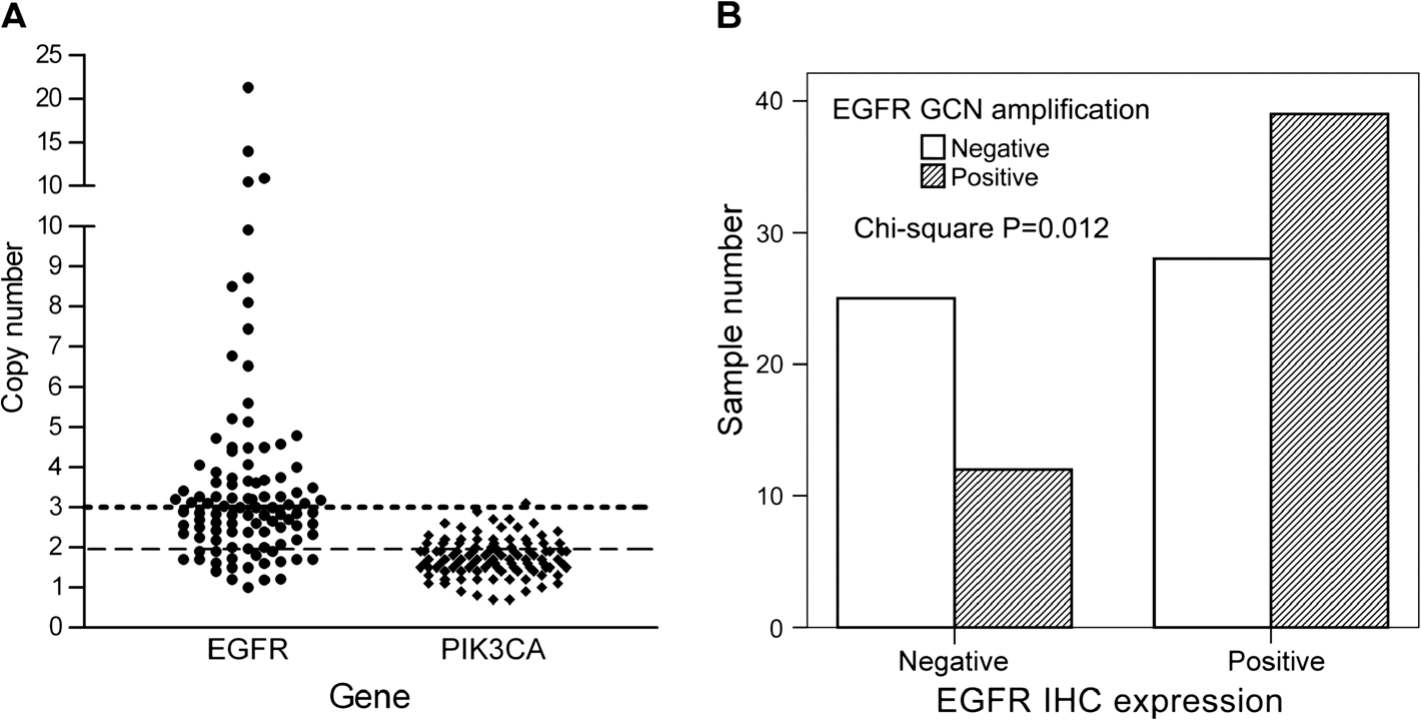
**Gene copy numbers of the samples.** (**A**) A scattergram of the *EGFR* or *PIK3CA* results are shown. Each dot represents the specific GCN of the individual specimen analyzed using real-time PCR and the C^-2∆tt^ method. Dots above the upper dotted line have copy number of more than 3. Dots above the lower dashed line have copy number of more than 2. (**B**) An association study of EGFR expression and GCN amplification is shown by the bar chart. The empty and solid bars represent negative or positive GCN amplification. The *P* level was assessed through Chi-Square analysis.

We next investigated GCNs using real-time PCR. Of 104 successfully analyzed samples for *EGFR*, 51 exhibited GCN amplification (49.0%; Figure 3A). Further investigation showed that of the 67 samples with EGFRwt expression, 39 had simultaneous *EGFR* GCN amplification. This was in comparison to the 12 samples (of a total 37) that had negative EGFRwt protein detection, demonstrating the expected correlation between these two factors (*P* = *0*.*012*; Figure 3B). In contrast, 26 of 105 samples (24.8%) displayed increased GCN for *PIK3CA*, including the only one with amplification.

For *PIK3CA* sequencing studies, segmental sequencing of the hotspot mutation site in exons 9 and 20 were successfully examined in 98 and 87 samples, respectively. Neither the G1624 nor the G1633 substitution was detected. Nevertheless, there were two samples bearing a point mutation at A3140, with one replaced by guanine and the other by thymine. Base substitution resulted in altered coding for arginine and leucine instead of histidine at the 1047 location of the catalytic domain. In conclusion, hotspot point mutations of *PIK3CA* only accounted for 2.3% of the OC samples.

### EGFRvIII expression correlates with tumor size and stage

We then evaluated the associations between EGFRvIII and other factors by grouping EGFRvIII into high-expression or negative/low-expression according to the IHC scores of 3+ and 4+ (31.5%) or 0/1+ and 2+ (25% negative, 43.5% low expression); Table 2). Within the 108 samples, 54 of them were recorded as stage 3/4 disease and 54 as stage 1/2 disease. High EGFRvIII expression levels were noted in 40.7% of stage 3/4 disease cases and in 22.2% of stage 1/2 disease cases. A significant association was observed between the expression of the truncated protein and disease stage (*P* < *0*.*001*). A similar observation was noted for the T but not N classifications (*P* < *0*.*001* and *P* = *0*.*071*, respectively).

**Table 2:** Association analyses of EGFRvIII expressing status with the other factors

		**EGFRvIII expression**		
	**Total**	**Negative or low**	**High**	***P*-value**
Staging				
T classification^#^	108			
1	38	35	3	<0.001
2	36	20	16	
3	6	4	2	
4	28	15	13	
N classification	108			
0	72	53	19	0.071
1	15	11	4	
2	21	10	11	
TNM stage	108			
1	30	29	1	<0.001
2	24	13	11	
3	13	10	3	
4	41	22	19	
Factors				
EGFR GCN amplification	104			
Negative	53	38	15	0.529
Positive	51	33	18	
EGFR expression	108			
Negative	39	27	12	1.000
Positive	69	47	22	
PIK3CA GCN increase	105		1.000	
Negative	79	53	26	
Positive	26	18	8	
PTEN expression	108			
Negative	53	41	12	0.064
Positive	55	33	22	
pAKT expression^#^	107			
Negative	14	11	3	0.543
Positive	93	63	30	

P-value was estimated using chi-square method or ^#^Fisher’s exact test.

We next focused on the interactions among EGFRvIII and other signaling pathway members. As shown in Table 2, high EGFRvIII expression levels were detected in 35.3% of the samples with *EGFR* GCN amplification and in 31.9% of those with EGFRwt protein expression (*P* = *0*.*529* and *P* = *1*.*000*, respectively). In addition, 40.0% of the 55 PTEN-positive samples showed high EGFRvIII expression levels compared with 22.6% of the PTEN-negative samples (*P* = *0*.*064*). The result was also not significant in *PIK3CA*. High expression of the variant protein was noted in 30.8% of the samples with increased *PIK3CA* GCN, comparing to 32.9% of those which were not increased (*P* = *0*.*1000*). Finally, high expression levels of the mutant receptor were observed in 32.3% of the 93 pAKT-positive and 21.4% of the pAKT-negative specimens (*P* = *0*.*543*). The analyses showed nonsignificant results for the association of EGFRvIII status and other biomarkers in the cascade.

### EGFRvIII and pAKT expression correlates with poor patient prognosis

**Figure 4:**
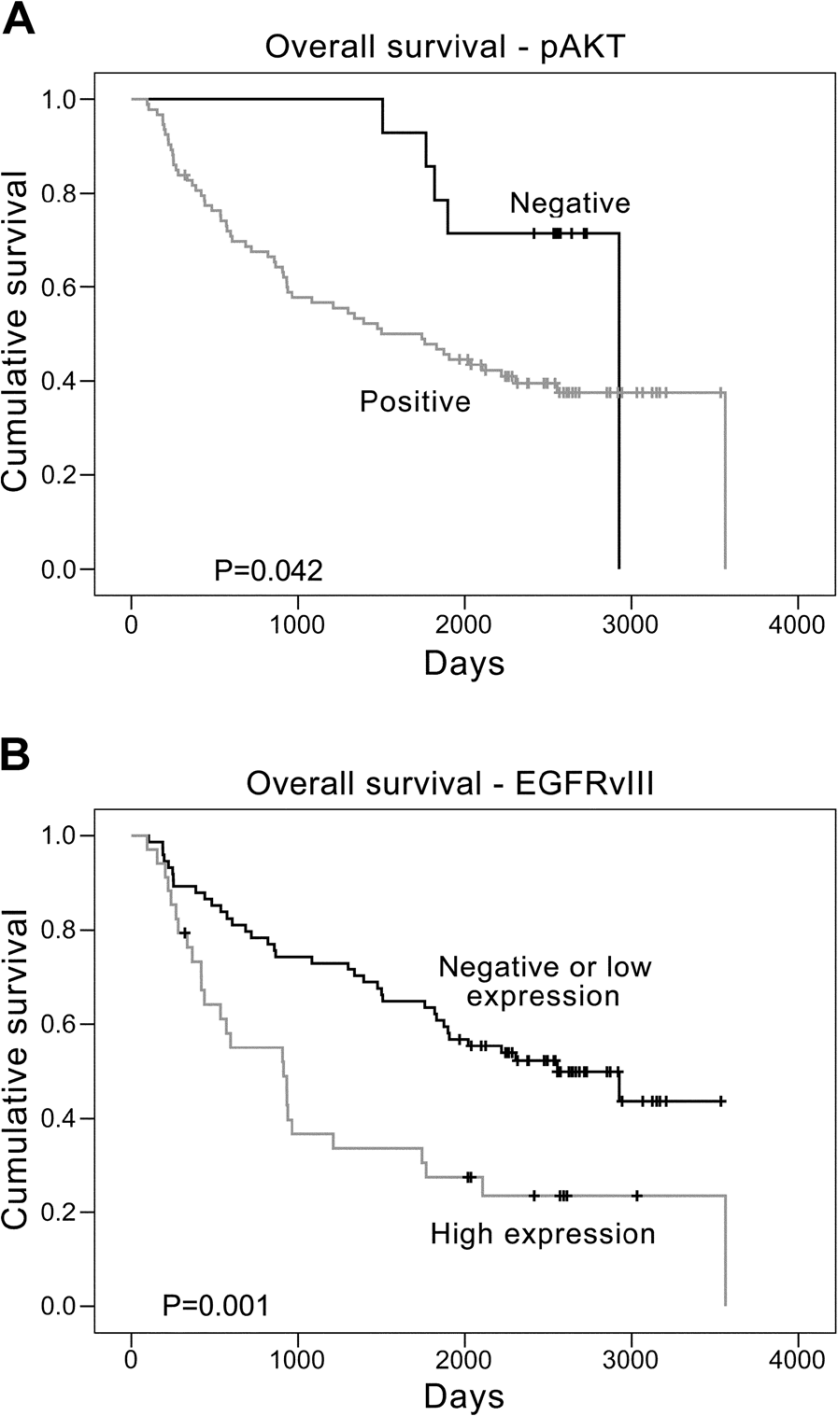
**The impact of pAKT and EGFRvIII on patient survival.** A representative Kaplan-Meier plot for overall survival based on the classification of (**A**) pAKT status, positive or negative, and (**B**) EGFRvIII expression, negative/low or high. The vertical tick marks indicate censored data, including patients who were alive at the final day or who were lost to follow up.

EGFR has been suggested to be a prognostic factor in HNC [26,27]. In our analyses, classification by PTEN status and EGFRwt protein expression and GCN were insufficient to show survival differences with their corresponding groups (*P* > *0*.*05* for all factors). In contrast, the survival curves for patients with different pAKT or EGFRvIII statuses showed significant differences (*P* = *0*.*042* and *P* = *0*.*001*, respectively; Figure 4A and 4B). We then studied the survival impact of multiple parameters including age, sex, history of unhealthy habits, and aberrant factors. The results of univariate analyses indicated that stage 4 disease and EGFRvIII and pAKT expression statuses were the applicable factors (*P* < *0*.*001*, *P* = *0*.*002* and *P* = *0*.*050*, respectively; Table 3). Both EGFRvIII and pAKT were then included in a stepwise regression with age, TNM stage, and habits. The result revealed that in addition to the disease stage, the expression levels of EGFRvIII and pAKT status were significant factors for patient survival after adjustment (*P* = *0*.*007*, *P* = *0*.*046*, and *P* = *0*.*049*, respectively).

**Table 3:** Univariate and multivariate analyses for the overall survival

		**Univariate analysis**		**Multivariate analysis**	
		**HR (95% CI)**	***P*-*value***	**HR (95% CI)**	***P*-value**
Age^#^	≤30	NA^§^		NA^§^	
	31-40	1 (reference)		1 (reference)	
	41-50	0.88 (0.43–1.81)	0.721	0.64 (0.25–1.62)	0.343
	51-60	0.67 (0.31–1.47)	0.318	0.83 (0.32–2.15)	0.705
	61-70	1.17 (0.52–2.66)	0.704	0.96 (0.30–3.08)	0.949
	≥71	0.51 (0.11–2.29)	0.376	0.52 (0.06–4.73)	0.564
Alcohol^#^	No	1 (reference)		1 (reference)	
	Yes	1.03 (0.62–1.74)	0.899	1.07 (0.54–2.11)	0.842
Smoking^#^	No	1 (reference)		1 (reference)	
	Yes	1.44 (0.71–2.93)	0.315	1.98 (0.79–4.97)	0.145
Betel nut^#^	No	1 (reference)		1 (reference)	
	Yes	1.64 (0.83–3.26)	0.156	1.00 (0.42–2.37)	0.999
TNM staging^#^	1	1 (reference)		1 (reference)	
	2	1.18 (0.50–2.78)	0.703	0.76 (0.26–2.23)	0.618
	3	2.35 (0.95–5.86)	0.066	2.16 (0.78–6.02)	0.140
	4	4.21 (2.12–8.40)	<0.001	3.53 (1.41–8.81)	0.007
EGFR	Negative	1 (reference)			
	Positive	1.19 (0.70–2.02)	0.516	NA	
EGFR GCN	Non-amplified	1 (reference)			
	Amplified	0.94 (0.57–1.57)	0.822	NA	
EGFRvIII^#^	Low	1 (reference)			
	High	2.27 (1.36–3.79)	0.002	1.98 (1.01–3.87)	0.046
PTEN	Negative	1 (reference)			
	Positive	1.26 (0.77–2.08)	0.359	NA	
pAKT^#^	Negative	1 (reference)			
	Positive	2.51 (1.00–6.27)	0.050	2.67 (1.01–7.07)	0.049

*CI* confidence interval, *HR* hazard ratio, *NA* not assessed.

^#^Factors included in multivariate analysis.

^**§**^Not assessed because the patient number was too small.

## Discussion

Understanding cancer biology is fundamental for specific target selection in developing drugs for modern oncology. Our study focused on the EGFR-PI3K cascade in OC, which demonstrated a high frequency of dysregulated factors. In addition to the frequently observed EGFRwt and GCN variations, EGFRvIII and the absence of PTEN were also notable. However, *PIK3CA* gene aberrations were rare in OC. Together, the pathway anomalies led to activated pAKT, which impacted patient survival. Moreover, our study reinforced the indispensable role of EGFR in OC, with EGFRvIII in particular being the major contributory element that influenced patient survival and outcome. To our knowledge, we are the first to report survival differences by EGFRvIII classification in OC.

The truncated variant EGFRvIII draws attention to the constitutive signaling activity, which is independent of ligand binding [28]. The actual mechanism of the production of this mutant receptor remains unknown. In a HNC study conducted by Sok, et al., EGFRvIII was exclusively expressed in coexistant with EGFRwt [11]. The different observation in our study may therefore suggest a distinguished feature specified to OC comparing to other HNC diseases, or rather a unique feature in betel-nut prevalent area. In fact, a breast cancer study has indicated that EGFRvIII expression is not a consequence of EGFR locus rearrangement or amplification but is rather due to alterative splicing events [29]. This notion was supported by our study and other brain tumor studies, as the appearance of EGFRvIII was not necessarily co-existed or co-related with EGFRwt protein expression or gene amplification [30,31]. In these cancer diseases, the probable indispensable roles of the mutant protein in tumorigenesis are therefore to be expected. Unlike in brain tumors, in which the impact of EGFRvIII on survival is known [32-34], its function in OC and other HNC are ambiguous, though frequently detected. In addition to our study, previous studies have failed to prove the determinant role of the truncated protein for survival outcome [27,35]. This failure may be due to the preceding reason and due to the different methods utilized in the investigations. In fact, accurately detecting the mutant protein is challenging because of interference from EGFRwt and the nearby non-tumor tissue. Different from the two studies utilizing RT-PCR, we used IHC staining because of its direct indication of the signal distribution. In addition, IHC possesses a specificity that is comparable to PCR over laser capture microdissected tissue [36]. The application of this method led us to demonstrate the crucial role of EGFRvIII. This result was not unexpected because of the association with disease stage, which has also been shown by Keller and colleagues [37].

In our studies, we found that the major subcellular distribution area of the variant protein was in the cytoplasm, whereas EGFRwt expression was detected at both the membrane and in the cytoplasm. This result was consistent with those observed in the human glioblastoma samples and in the breast cancer samples [36,38]. The definite causes leading to the distinct phenomenon are not yet clear. It has been suggested that trivial differences in signaling and functioning distinguish EGFRvIII and its wild-type counterpart. One possible example is the defective downregulation of the truncated receptor due to the hypophosphorylation of residue Y1045, leading to escape from ubiquitination by c-Cbl [39]. Another example was the demonstration of a large intracellular pool of EGFRvIII functioning with Src to reduce the glucose dependency when relocalizing to the mitochondria [40]. Taken together, these studies along with our data support the role of cytoplasmic EGFRvIII in tumorigenesis. Therefore, further research to elucidate the mechanism of intracellular redistribution is imperative.

Though positive correlation was shown between the EGFR expression and the GCN amplification, we found that they were not completely overlapped. In recent reports, discrepancies have been noted between these two factors in OC [25,41]. The exact mechanisms in the samples that had controversies within the two factors remain unclear. Regarding the protein producing process, the accurate regulatory mechanism of transcription and translation from the *EGFR* coding gene is not fully understood. Complicated modulation against *EGFR* gene transcription was noted, for example, that the regulation mechanism depends on the length of a CA repeat in intron 1 [42]. In addition, Wheeler and colleagues found despite of correlation with *EGFR* gene amplification status and the protein levels, the level of EGFR mRNA was not associated to both factors [27]. This indicated post-translational regulation was at least in part crucial to the protein expression. Therefore, it was not surprising that some samples have positive EGFR protein expression without GCN amplification, and vice versa.

Recent investigations have emphasized the role of PI3K in HNC [43]. Our study of the *PIK3CA* gene, however, indicated that GCN amplification or hotspot point mutations were rare. This finding was in contrast to several reports that highlighted the frequent aberrations of the gene itself. Examples of such reports include a study that found that 34.8% of OC samples exhibit high GCN amplifications [44] and another showing that 11% of HNC samples carry hotspot point mutations [11]. Nevertheless, contradictory results were also noted in the OC study conducted by Kozaki and colleagues [45]. In the 108 OC primary tumor samples analyzed, 16.7% of those showed altered copy number for a 1.3 ~ 3.4-fold increase, and four samples were detected of bearing hotspot point mutations. Taken together with our study, these data suggest that GCN variations of *PIK3CA* encoding areas were not as significant as *EGFR* alterations. Finally, the possibility that the discrepancy in these observations is due to population differences should be further examined.

The pAKT expression levels in our samples were high and correlated with patient survival. As the preferred signaling protein, it was unexpected that its associations with EGFRvIII or EGFRwt (data not shown) expression were not significant [46]. One reasonable potential explanation is the interruption of the other regulatory factors as compensation. For instance, PTEN is known to be a negative modulator of this pathway. Because AKT, and likely STAT3 [47], are oppositely regulated by EGFRvIII and PTEN, its activation was thus speculated as a result of feedback reaction. In addition, further studies have uncovered alternative routes that may be responsible for tumorigenesis by the truncated protein, such as through CXCR4 and cyclooxygenase-2 [48,49]. These proteins bypass the cascade, attenuating the dependency of the AKT pathway without dispelling EGFRvIII tumorigenic impact.

## Conclusion

We demonstrated the indispensible roles of pAKT and EGFRvIII in OC, which likely act as the determinant factors for patient survival. The data suggest that pAKT and EGFRvIII could be used as prognostic markers, and a validation of our findings is warranted. Although not studied here, the fact that frequent aberrations in EGFR-PI3K-AKT pathway lead to resistance to EGFR targeting therapy should be further investigated in OC [11,50,51]. Finally, our results lead to the rationale for future clinical investigations of the specific inhibitors that have already shown benefit for tumor control [52,53].

## Abbreviations

EGFR: Epidermal growth factor receptor; EGFRvIII: Epidermal growth factor receptor variant III; EGFRwt: Epidermal growth factor receptor wild type; FFPE: Formalin-fixed paraffin-embedded; GCN: Gene copy number; HNC: Head and neck cancer; IHC: Immuno histo chemical; OC: Oral cancer; PCR: Polymerase chain reaction; PI3K: Phosphoinositide-3-kinas; PTEN: Phosphatase and tensin homolog; pAKT: Phosphorylated AKT.

## Competing interests

The authors declare that they have no competing interests

## Authors’ contributions

KYC participated in the interpretation of the data and drafted the manuscript. SYT interpreted the data. SHC and CYY performed the sample collection. SWC and BFC performed the experiment. HCW, HLF, and KJL performed the data collection. HHT performed the statistical analysis. CJY and SYL performed the final approval of the manuscript. PJL and CKT participated in the design of the study. JYC conceived of the study, and participated in its design and coordination and helped to draft the manuscript. All authors read and approved the final manuscript.
